# Prenatal diagnosis and molecular cytogenetic analysis of a *de novo* isodicentric chromosome 18

**DOI:** 10.4103/0256-4947.70578

**Published:** 2010

**Authors:** Yanliang Zhang, Yong Dai, Jinghui Ren, Linqian Wang

**Affiliations:** a From The Clinical Medical Research Center, Second Clinical Medical College (Shenzhen People’s Hospital), Jinan University, Guangdong Province, China; b From The Hereditary Disease and Prenatal Diagnosis Center, Second Clinical Medical College (Shenzhen People’s Hospital), Jinan University, Guangdong Province, China

## Abstract

Isodicentric chromosome 18 [idic(18)] is rare structural aberration. We report on a prenatal case described by conventional and molecular cytogenetic analyses. The sonography at 24 weeks of gestation revealed multiple fetal anomalies; radial aplasia and ventricular septal defect were significant features. Routine karyotyping showed a derivative chromosome replacing one normal chromosome 18. The parental karyotypes were normal, indicating that the derivative chromosome was *de novo*. Array comparative genomic hybridization (array-CGH) revealed 18p11.21→qter duplication and 18p11.21→pter deletion for genomic DNA of the fetus. The breakpoint was located at 18p11.21 (between 12104527 bp and 12145199 bp from the telomere of 18p). Thus, the derivative chromosome was ascertained as idic(18)(qter→p11.21::p11.21→qter). Fluorescent in situ hybridization (FISH) confirmed that the derivative chromosome was idic(18). Our report describes a rare isodicentric chromosome 18 and demonstrates that array-CGH is a useful complementary tool to cytogenetic analysis for reliable identifying derivative chromosome.

Trisomy 18 is a common chromosomal disorder resulting in well-known dysmorphic features and organ malformations. Isochromosome 18 [i(18q) or i(18p)], however, is rare cytogenetic aberration. Because the dicentric pattern is difficult to establish using conventional banding analysis alone, the report of isodicentric chromosome 18 [idic(18)] is more rare.^1^ We present a fetus with a previously undescribed isodicentric chromosome 18 [idic(18)(qter→p11.21::p11.21→qter)] characterized by array comparative genomic hybridization (array-CGH) and fluorescent in situ hybridization (FISH).

## CASE

**Figure 1:**
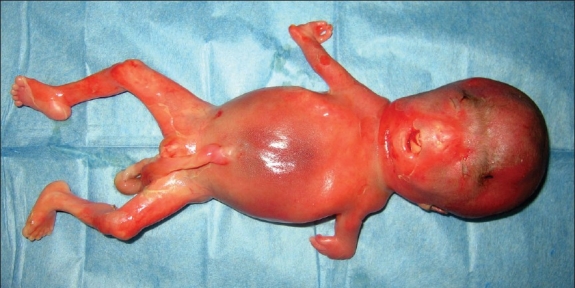
Photograph of the fetus after termination at 27 weeks of gestation.

A 28-year-old gravida 3 para 0 Chinese woman was referred to our hospital at 24 weeks of gestation because sonography showed multiple fetal anomalies, including bilateral absent radii and short humeri, deformed hands, a right cross-foot, a ventricular septal defect, bilateral choroid plexus cysts, a thickened neck and nape soft tissue. The pregnancy was uneventful. The woman and her husband were healthy and non-consanguineous. There was no family history of congenital malformations. She denied any exposure to alcohol, teratogenic agents, irradiation or infectious diseases during the pregnancy. The analysis of cultured amniotic fluid cells yielded 100% of metaphases with chromosomal abnormality involving chromosome 18. The parental karyotypes were normal. Based on the sonographic findings and the unbalanced chromosomal aberration, the pregnancy was terminated at 27 weeks of gestation. At termination, the fetus was phenotypically male measuring 32 cm in length. Physical examination revealed bilateral valgus palms, bilateral short upper limbs, right cross-foot, and left pes valgus (**Figure 1**). Necropsy further confirmed bilateral absent radii, bilateral ulnas length 2.5 cm, left humerus length 2.0 cm, right humerus length 3.0 cm, bilateral deformed articulatio capitis humeri, articulatio cubiti, articulatio carpi, articular genu and articulatio talocruralis, angiocavernoma in the cerebellum, ventricular septal defect, aorta diameter 0.4 cm, and pulmonary artery diameter 0.3 cm. Other internal organs seemed normal on gross appearance.

**Figure 2:**
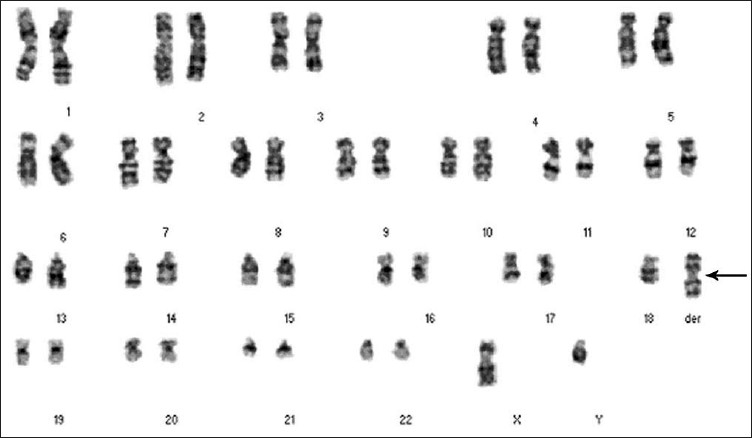
G-banded karyotype of the proband.

**Figure 3:**
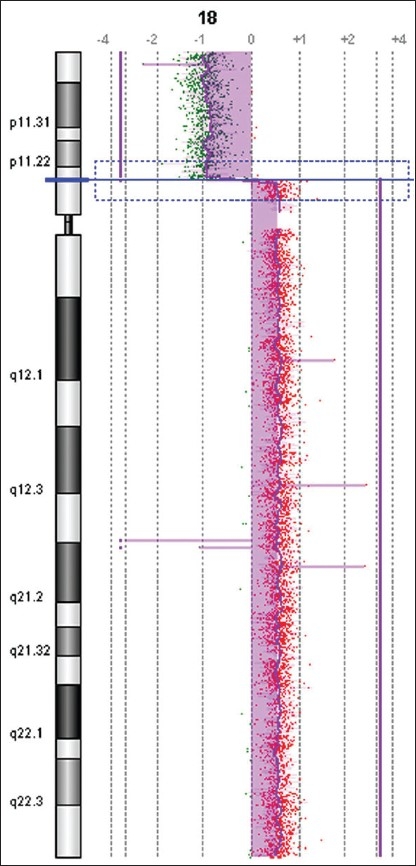
Array comparative genomic hybridization ratio profile of the derivative chromosome 18.

G-banding analysis on cultured amniotic cells showed that one chromosome 18 was replaced by a derivative chromosome (**Figure 2**). The origin and architecture of the derivative chromosome were unclear. Both parents had normal karyotypes, indicating a *de novo* origin of the derivative chromosome 18. Array-CGH revealed 18p11.21→qter duplication and 18p11.21→pter deletion for genomic DNA of the fetus, the breakpoint was located at 18p11.21 (between 12 104 527 bp and 12 145 199 bp from the telomere of 18p), which indicated that the derivative chromosome was idic(18)(qter→p11.21::p11.21→qter) (**Figure 3**). FISH analysis using a satellite centromere-specific probe for chromosome 18 (D18Z1), whole chromosome painting probe for chromosome 18 (WCP18), 18q/18p telomere-specific probes (D18S1390 and D18S552) (Cytocell, UK) confirmed that the derivative chromosome 18 was idic(18) (**Figure 4A to 4D**). Written informed consent was obtained from the patient’s parents. This study was performed according to the guidelines of Shenzhen People’s Hospital, which abides by the Helsinki Declaration on ethical principles for medical research involving human subjects.

**Figure 4A:**
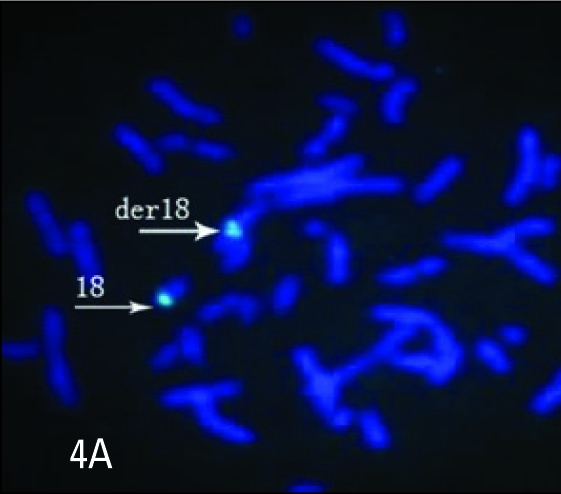
A partial metaphase spread hybridized with a chromosome 18 centromere probe (green) showing two signals in derivative chromosome 18 and one signal in normal chromosome 18.

**Figure 4B:**
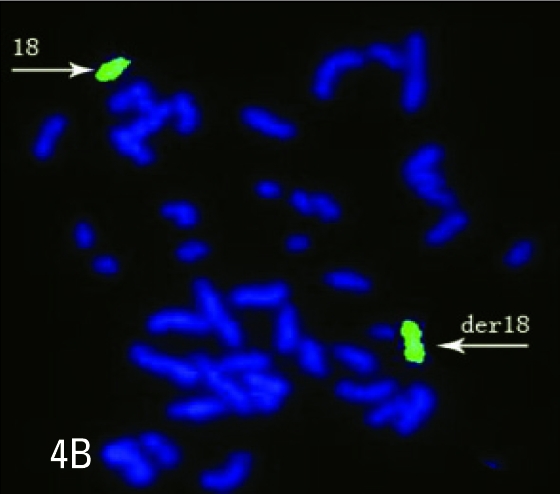
A metaphase spread hybridized with a whole chromosome 18 library (green) identifying the derivative chromosome as being composed of chromosome 18 material.

**Figure 4C:**
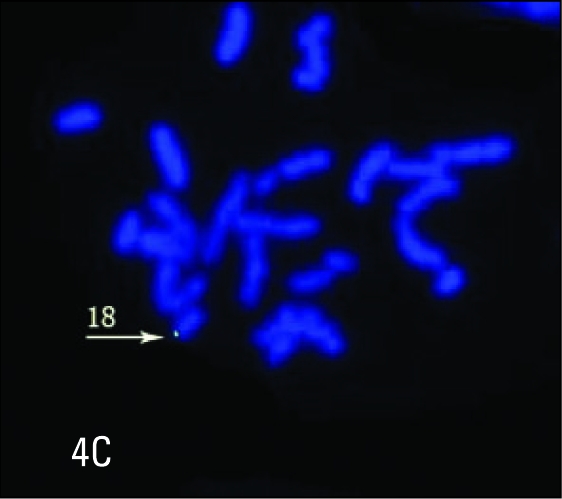
A metaphase spread hybridized with 18p subtelomere probe (green) showing only one normal chromosome 18 with a p-telomere signal at one end.

**Figure 4D:**
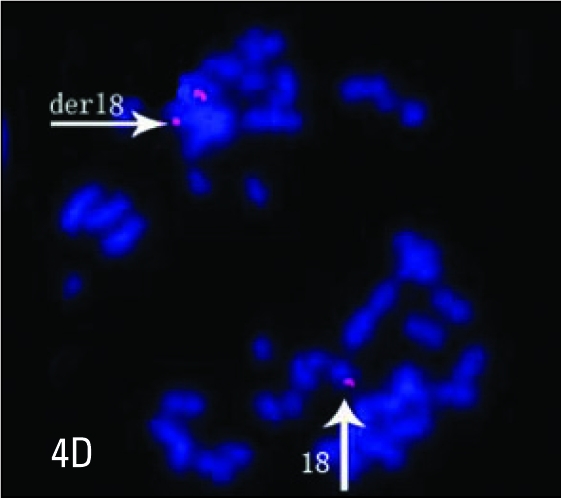
A metaphase spread hybridized with 18q subtelomere probe (orange) showing one derivative chromosome 18 with q-telomere signals at both ends and one normal chromosome 18 with q-telomere signal at one end.

## DISCUSSION

The current standard technique for detecting chromosomal abnormalities involves metaphase analysis of cultured cells. Due to limited resolution, only large aberrations (> 3 Mb) and translocations can be identified by conventional cytogenetic techniques. Therefore, in this case, G-banding analysis could not identify the origin and architecture of the derivative chromosome. Only by applying array-CGH could the derivative chromosome be ascertained to be idic(18)(qter→p11.21::p11.21→qter), indicating that array-CGH is a useful tool for describing derivative chromosome.

Isochromosomes are supernumerary marker chromosomes made up of two copies of the same arm of a chromosome containing 1 to 2 centromeres, so that they form a mirror image of each other.^2^ Dicentric autosomes are not common in humans because they often result in partial trisomy and partial monosomy status. There are two mechanisms by which an isodicentric chromosome can be formed. One is an isolocal U-shape sister chromatid exchange (isolocal break in two chromatids and rejoining of broken ends) in either the S or G2 phase.^3^ The other is a chromosomal deletion during the G1 phase and U-shape rejoining of the broken ends of the chromatid after replication of the deleted chromosome.^4^ Both mechanisms will result in a symmetrical isodicentric chromosome. Recently, a new type of asymmetrical pseudoisodicentric chromosome derived from non-isolocal sister chromatid breaks of a parental homolog has been reported.^5^

It has been noted that numerical and structural abnormalities of chromosome 18 usually produce severe phenotypic abnormalities. The isochromosome 18q [i(18q)] often demonstrates characteristics of pure trisomy 18 owing to three copies of 18q as well as monosomy 18p due to missing one copy of 18p. The typical phenotype of i(18q) is very comparable to complete trisomy 18 and includes severe growth retardation, occipital prominence, low set ears, downward-slanting palpebral fissures, overlapping flexed fingers, rocker-bottom feet, congenital heart defects, and skeletal abnormalities. The monosomy 18p (18p-) often results in psychomotor retardation, short webbed neck, low posterior hairline, hypospadias, male genital anomalies and holoprosencephaly.^6^,^7^ The present fetus who had 18p11.21→qter duplication and 18p11.21→pter deletion demonstrated typical features of trisomy 18, including ventricular septal defect, limbs malformations and skeletal abnormalities. However, the cardinal features of 18p- were not present. This may be because 18p- features are less specific and camouflaged by the more drastic manifestations of trisomy 18q or a significant amount of short arm material (18p10→p11.21) is present. Furthermore, patients with a monocentric i(18q) usually have manifestations of both trisomy 18 and the 18p- syndrome; however, if the isochromosome 18 is dicentric, only the characteristics of trisomy 18 are likely to be exhibited.^8^ Therefore, the redundant centromere presented in our case might influence the presence of phenotypes of 18p-. Wulfsberg et al suggested that Turner-like features associated with 18p- may be determined by monosomy for 18p11.^9^ Our case, in whom 18p10→p11.21 is present, lacked Turner-like features, which supports the conclusion of Wulfsberg et al, and we postulate that 18p10→p11.21 may be the critical region for Turner-like features associated with 18p-. Our case had severe limb malformation. A dosage imbalance of SALL3 located on 18q may be responsible for the radial defects.^10^ Our case also has angiocavernoma in the cerebellum, which to our knowledge, has not been reported in cases with idic(18) or i(18q).

In conclusion, this report describes a rare *de novo* isodicentric chromosome 18 and demonstrates that array-CGH is a useful complementary tool for cytogenetic analysis in reliably describing a derivative chromosome. There might still be some technical and financial problems using the arrays in routine diagnostics, but array-CGH has already become an important tool for the diagnosis of genetic disease in clinical practice.
